# Editorial: Biosurfactants: From renewable resources to innovative applications

**DOI:** 10.3389/fbioe.2022.988646

**Published:** 2022-08-11

**Authors:** Murat Ozdal, Tomasz Janek, Surekha K. Satpute

**Affiliations:** ^1^ Department of Biology, Science Faculty, Ataturk University, Erzurum, Turkey; ^2^ Department of Biotechnology and Food Microbiology, Wrocław University of Environmental and Life Sciences, Wrocław, Poland; ^3^ Department of Microbiology, Savitribai Phule Pune University, Pune, India

**Keywords:** biosurfactant, applications, pathogens, fermentation, nanoparticle

The Research Topic entitled “*Biosurfactants: From Renewable Resources to Innovative Applications*” has meticulously contributed original research and review articles towards pioneering applications of biosurfactant/s (BSs) in plentiful areas like medical, pharmaceutical, agriculture, bioremediation, waste-water treatment, etc.

The multifarious functional potential, phase behaviour, huge structural diversity, biodegradable and eco-friendly nature of BSs offer enormous opportunities to explore them for innovative applications. Emphasis on employment of appropriate, precise, accurate methodologies are mandatory to achieve effective isolation, production, extraction and characterization of BSs. Continuous efforts are desirable in the BSs fermentation processes to develop commercially sound technology. Nevertheless, challenges like requirement of desirable microbial strains, growth substrates, high monetary inputs, downstream processing etc. hinder the economical production of BS. Legalizing the BSs production technology through vigorous experimental conditions are indispensable. Genome mining studies offer new-fangled avenues of BSs producers for benefit of mankind.

The various articles published under this Research Topic deliver guiding principles and vital operating protocols to elevate the production of BSs from diverse microorganisms. Contributors have portrayed putative ground-breaking applications of BSs in varied industries. Review article of Salek et al. showcased extensive uses of rhamnolipids (RL), sophorolipids (SL), mannosylerythritol lipids (MEL) and cellobiose lipids (CL) in medical, agriculture, bioremediation, food, detergent, household-care agents and clean-up strategies.

Regardless of the empathetic knowledge of BS molecule; there is a prerequisite to investigate the process at the bioreactors level to establish economical level production. Beck et al. reported MEL fermentation from *Moesziomyces aphidis* in a bioreactor with monitoring of kinetics for process parameters (substrate consumption rates, product formation rates). An enhanced level of biomass along with yield of MEL has been achieved successfully through an exponential fed-batch approach. Another research group of Oraby et al. has optimized fermentation process for CL from Ustilaginaceae sp. using modelling and techno-economic studies.

The purification process of microbial metabolites has a prodigious impact on monetary inputs and further their applicability. Till today, plentiful procedures have been employed to produce and purify BS. Conventionally, the freeze-drying technique is employed to produce lipopeptides from *Bacillus subtilis*. Vassaux et al. have operated the spray-drying process to purify three lipopeptides - surfactin, mycosubtilin and plipastatin without affecting their antimicrobial and surfactant potential. Therefore, the proposed innovative technique can replace the conventional freeze-drying steps from an industrial perspective.

Indigenous microbes dwelling in oil/hydrocarbon/metal-polluted sites attain incredible potential to utilize those resources as compared to non-native entities. The genomic analysis of BS producing microbes from such habitats is imperious to improve future production processes and discovery of bioactive compounds of industrial interest. Yasmin et al. reported genome studies of BS producing *B. subtilis* strains. Researches have revealed protein-coding genes responsible for virulence, metal or multidrug resistance, flagella assembly, biosynthesis of secondary metabolites along with their role in emulsification, utilization and degradation of oil. The distinctness in gene operons and their involvement in the biosynthesis of surfactin along with their efficiency in degradation of oil has been explained in this article.

Removal of inorganic-based contaminants from soils and water bodies is extremely challenging. The existing remediation techniques are insufficient to manage and remove the pollutants completely. Nanoparticles (NPs), due to their extraordinary physico-chemical and functional properties have been utilized for remediation purposes. Moura et al. have demonstrated an attractive and low-cost green approach for remediation of contaminated water. Researchers have presented the functionalization of zero-valent iron (nZVI) with RL to remove inorganic pollutants from simulated groundwater. The role of RL in reducing the aggregation tendency of NP and removal of nitrate form contaminated water has been explained deeply.

BSs pose their candidature to deal with dreaded diseases like cancer. Till today, several studies have been reported for anticancer activity of RL. However, few studies have included triple-negative breast cancer. Additionally, the mechanistic role of RLs detailing molecular aspects has not been revealed completely. Mishra et al. have demonstrated noticeable anticancer activity of *Pseudomonas aeruginosa* origin RL against breast cancer cell lines. The radical scavenging activity of RL affects the cell line through the inhibition of p38 MAPKs. Targeting p38 for anticancer therapy is imperative due to its involvement in signalling pathways. Around 12 different congeners have been explored for anticancer activity of RL.

Worldwide, the agriculture sector is under continuous coercion to enhance productivity and meet the ever-rising food demands of the escalating population. The various factors (climate change, pesticide resistance etc.) lead to the emergence of pathogens in plants and reduce crop productivity. Additionally, humans are adversely impacting the environment through the use of enormous amounts of agrochemicals and intensive agronomic practices. Therefore, it is obligatory to develop an eco-friendly sustainable approach to deal the challenges. Karamchandani et al. has demonstrated antimicrobial activities of chitosan, chitosan NPs and BS singly and in combinations against selected phytopathogens. This work has led a strong hope to develop nano-formulation for sustainable and green agriculture. Like bacteria, filamentous fungi also proficiently produce several fungicidal compounds including BSs. Piegza et al. have used *Trichoderma citrinoviride* for production of BS in media accompanied by lytic enzymes inducers. Biocontrol potential of fungal derived BS has been demonstrated in this research article.

In summary, the valuable research presented in this thematic issue clearly demonstrated that breakthrough approaches are mandatory in the isolation, extraction and recovery of BS from various microbial sources. Selection of suitable microbial producers along with cheap or renewable agro-industrial substrates under precise optimizing fermentation parameters could lead not only great success at large-scale but also an economical production of BS. Further, BSs can be used singly or in combinations due to their functional potential in defeating dreaded pathogens or against cancer. Elucidation of mechanisms at biochemical, cellular levels along with preclinical or nonclinical studies is mandatory to broaden the applications of BSs. Information on the structure and function relationship of pure and/or a mixture of BSs would be undoubtedly obliging to recommend their many more extraordinary applications. Overall, the considerate knowledge about the innovative production technology, detailed physicochemical properties, innovative applications, molecular level studies of BSs have been contributed magnificently by the scientific fraternity.

